# Hydration Monitoring in the Long-Term Prophylaxis of Nephrocalcinosis
*Paper presented at a meeting of the South West Urologists Club


**Published:** 1991-03

**Authors:** N. M. Goble

**Affiliations:** Honorary Clinical Tutor in Urology, University of Bristol


### Hydration Monitoring in the Long-term Prophylaxis of Nephrocalcinosis*

**N.M. Goble**
Honorary Clinical Tutor in Urology, University of Bristol

### INTRODUCTION

Increasing fluid intake in order to reduce urine concentration has long been regarded as the single most important factor in preventing recurrent nephrocalcinosis.[1] The aim of this therapy is to reduce concentration below the upper limit of metastability for the solubility of stone forming salts. Pak et al[2] quantitatively assessed this potential, and concluded that high fluid therapy reduces the propensity for crystallisation of calcium salts. This objective evidence for the beneficial role of high fluid therapy was clinically demonstrated by Frank and de Vries[3] who by educating a community on these potential benefits were able to reduce the occurrence of stone disease by a factor of 10 when compared to a control community. Similarly, Robertson et al[4] identified low urine volume as the single most significant risk factor in calcium stone disease. Maintaining adequate fluid intake is, however, a difficult discipline for the patient to learn and the amount of fluid required can vary considerably, dependent upon ambient temperature and activity. Advising a fixed fluid intake over a 24hr period is, therefore, somewhat arbitrary as the amount of urine produced in response can vary widely. Ideally patients should adjust their fluid intake according to urine concentration. In conjunction with Gyrus Medical Ltd, a self-care advice *(Urimho)* was developed to facilitate compliance monitoring. The evaluation and development of this device is herein presented.

### PATIENTS AND METHODS

72 patients with known stone disease previously treated at Southmead Hospital and Bristol Royal Infirmary were requested to perform a water drinking test at home following a period of overnight dehydration. During the test patients consumed 200mls/20min of water and collected 10ml samples of all urine produced at each voiding during the test period. The first 27 patient samples were then analysed using refractometry and electrical conductivity. The ***Urimho*** device was then calibrated and tested on the remaining samples. Results were analysed using linear regression techniques. The 99.9% confidence interval for predicting the therapeutic threshold urine concentration at a specific gravity (SG) of 1.015[5,6] was then calculated. The range of urinary conductivity was then divided into 5 segments depending on the relationship to this threshold; two high (red scale), one at the threshold (yellow) and two low (green). A field evaluation on 22 patients with stone disease was then performed to establish accuracy, reliability and ease of use in the home environment. Patients were asked to consume their normal volume of fluids and not to comply with the remedial measures advised on the basis of the display reading. Otherwise they used the device at home according to instructions given in the user's manual. They collected numbered samples of urine voided during the test and recorded their display results. These samples were then analysed under laboratory conditions and the results compared.

### RESULTS

70 patients completed the test resulting in 466 urine samples. The overall relationship of SG to urine conductivity in the range 0–20mS was linear, r=0.94. The 99.9% confidence interval for the population mean of conductivity readings representing a urinary SG of 1.015 was 11.725–12.754mS. The scale of the ***Urimho*** device was then divided into 5 segments: <7.0mS, 7.0–10.5mS, 10.5–14.0mS, 14.0–17.13mS and >17.3mS. These segments represent, in terms of SG; <1.0083, 1.0083–1.0127, 1.0127–1.0173, 1.0173–1.0213 and >1.0213. These segments were coloured green 2, green 1, yellow, red 1 and red 2 respectively. The results of the field evaluation demonstrated mean SG results which correlated very well with the display readings, r=0.99. Only 8 (10%) of tests of the 75 performed in the laboratory differed by one display range from the lay user results. In only one test (1%) did this demonstrate a false negative result which would affect the remedial action according to the display interpretation given in the user's manual. The most notable feature of these results was that 56% of readings were in the red range.

*Paper presented at a meeting of the South West Urologists Club

### DISCUSSION

Adequate patient compliance with hydration therapy in the prevention of recurrent stones poses a considerable problem, well demonstrated by Cadoff et al.[7] The field study on the ***Urimho*** supports this concern, in that 56% of patients in the study demonstrated urine concentrations well in excess of the therapeutic threshold, despite the fact that all the patients had been advised on the benefits of high fluid intake. Without adequate feedback on progress, patients do appear to find this a difficult discipline. The ***Urimho*** provides this feedback thereby promoting patient compliance, a process enhanced by the use of a compliance chart for follow up examination by their urologist. Given that 60–80% of patients will develop a recurrence without adequate remedial measures, the use of the ***Urimho*** device offers considerable potential in reducing this recurrence rate.

### REFERENCES

1. EMBON, O.M., ROSE, G.A. and ROSENBAUM, T. (1990). Chronic dehydration stone disease. *Br. J. Urol.*, **66,** 357–362.
2. PAK, C.Y.C., SAKHAEE, K., CROWTHER, C. et al. (1980). Evidence justifying a high fluid intake in treatment of nephrolithiasis. *Ann. Int. Med.*, **93(1),** 36–39.
3. FRANK, M. and De VRIES, A. (1966). Prevention of urolithiasis. *Arch. Environ. Health.*, **13,** 625–630.
4. ROBERTSON, W.G., PEACOCK, M., HEYBURN, P.J. et al. (1981). Risk factors in calcium stone disease. In *Urinary Calculus.* ed. Brockis, J.G. and Finlayson, B. pp. 265–273. Littleton: PSG Publishing Co.
5. REINECKE, F., BURCHARDT, P. and KALLISTRATOS, G. (1976). Experience with long-term prophylaxis of kidney stones. In *Urolithaisis Research.* ed. Fleisch, H., Robertson, W.G., Smith, L.H. and Vahlensieck, W. pp 557–559. New York and London: Plenum Press.
6. LUTZEYER, W. and HERING, F. (1985). Drug therapy of urinary calculi and prevention of recurrence. In *Urolithaisis * Therapy * Prevention.* ed. Schneider, H-J. pp 2–5. Berlin, Heidelberg, New York and Tokyo: Springer-Verlag.
7. CADOFF, R.E., DRACH, G.W. and Le BOUTON, J. (1988). Specific gravity test strips used in monitoring urine concentrations of urolithiasis patients. *J. Urol.* **139,** 323–325.

